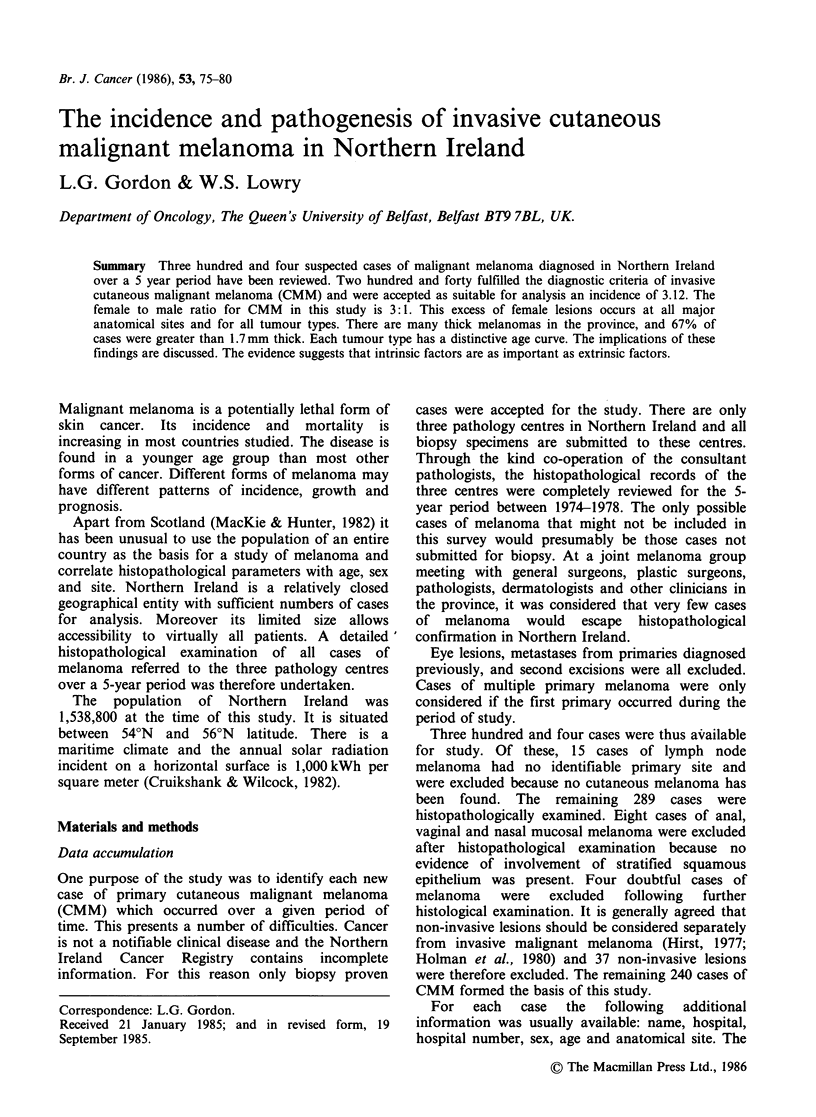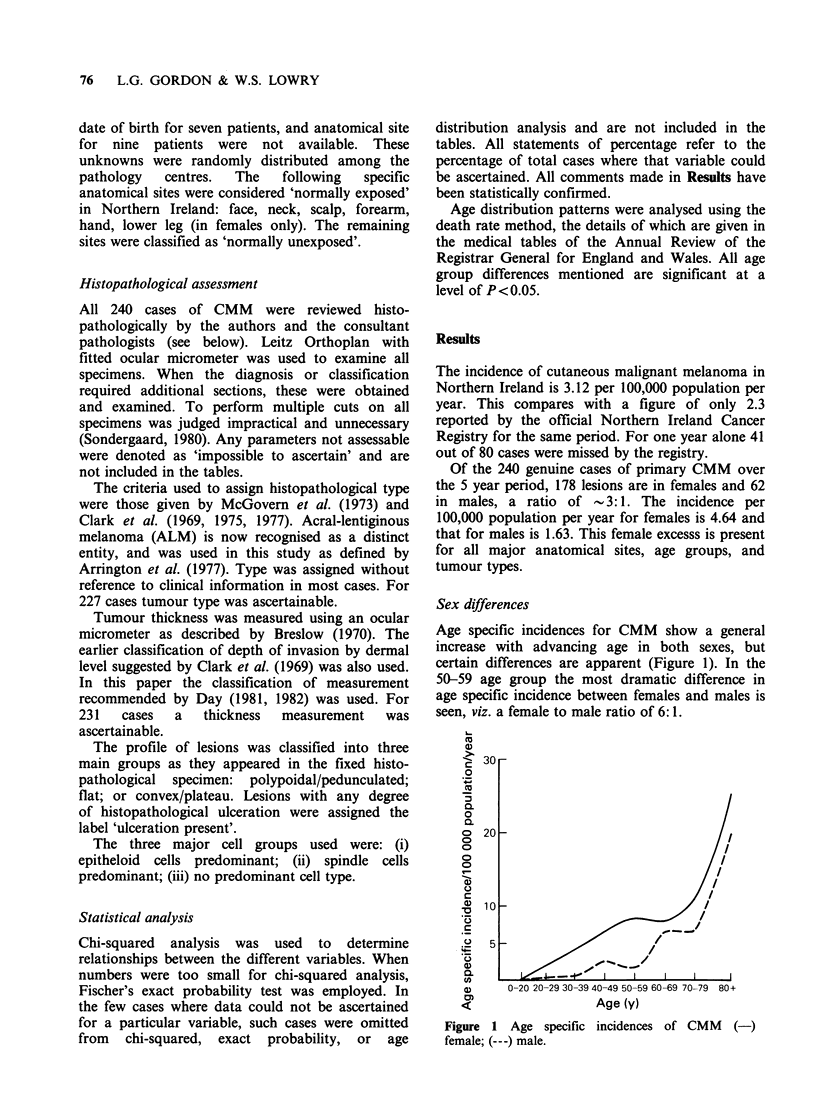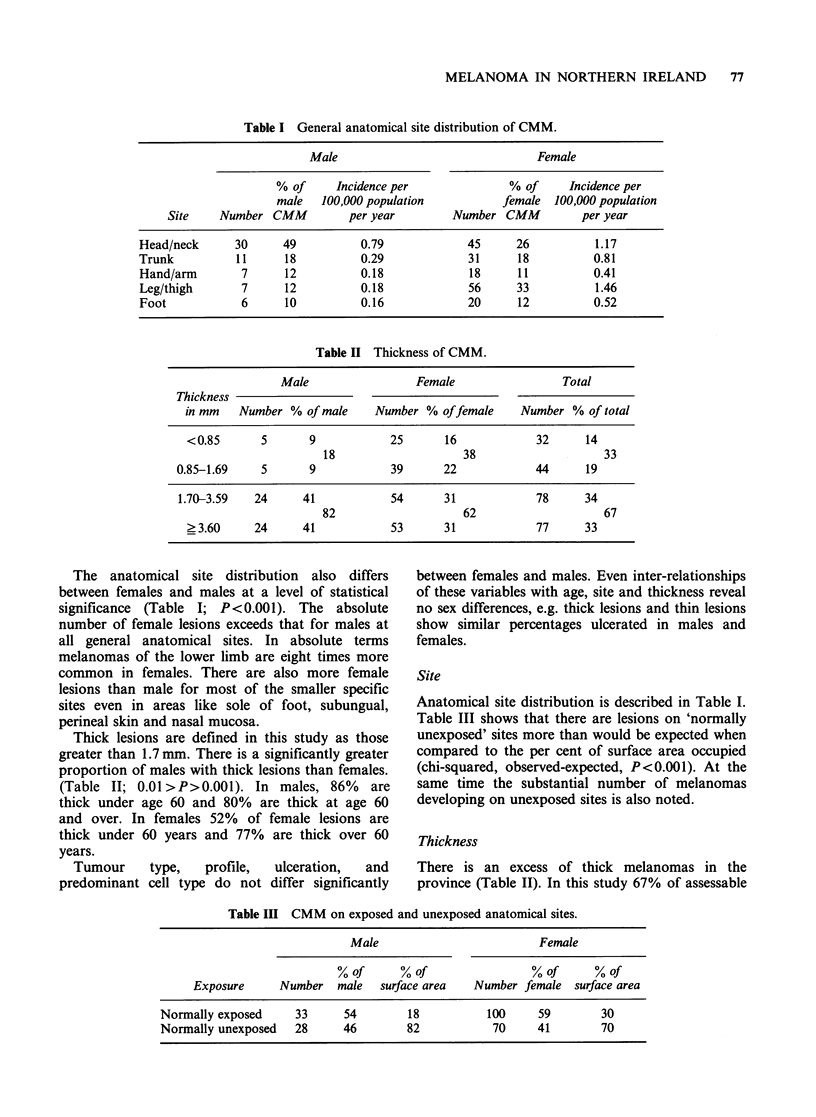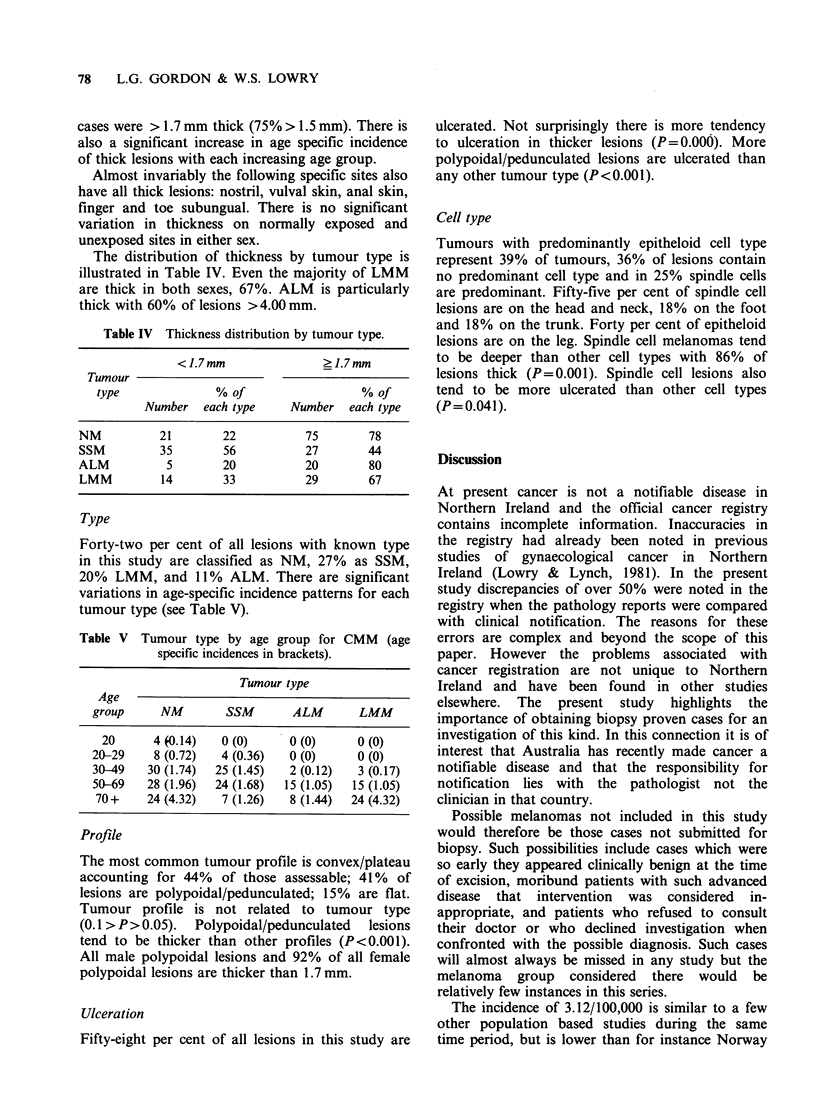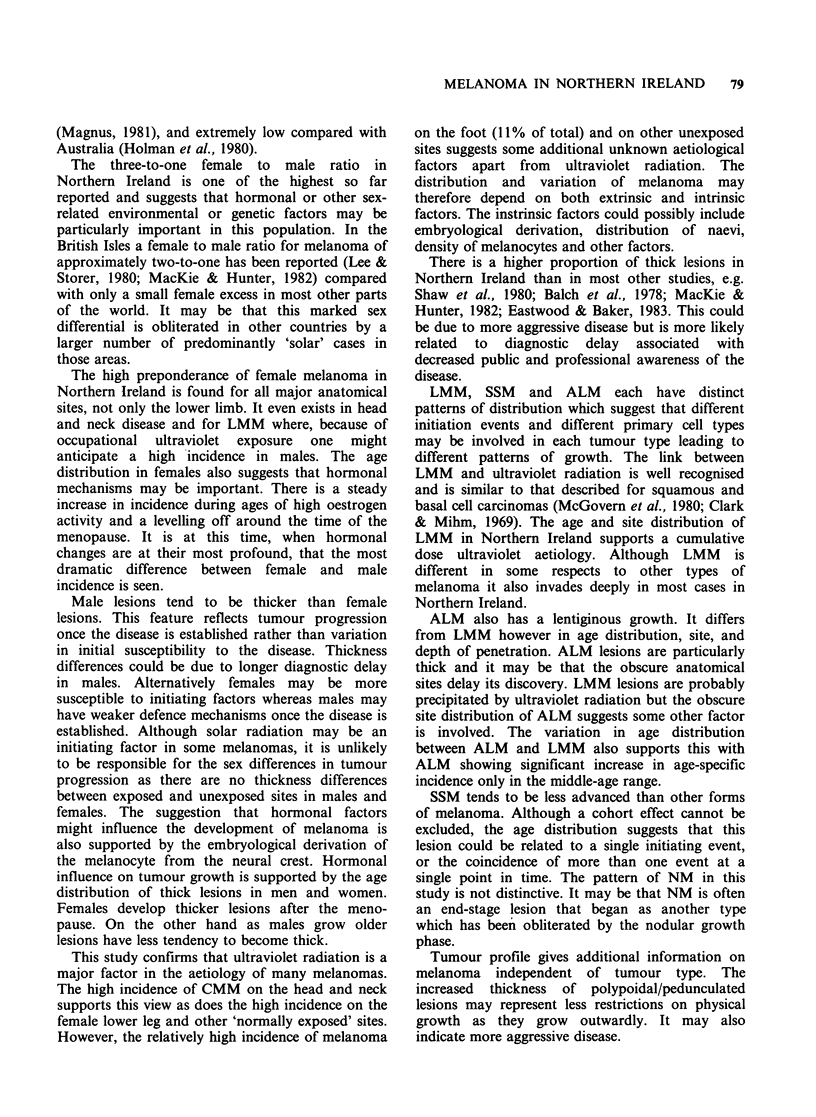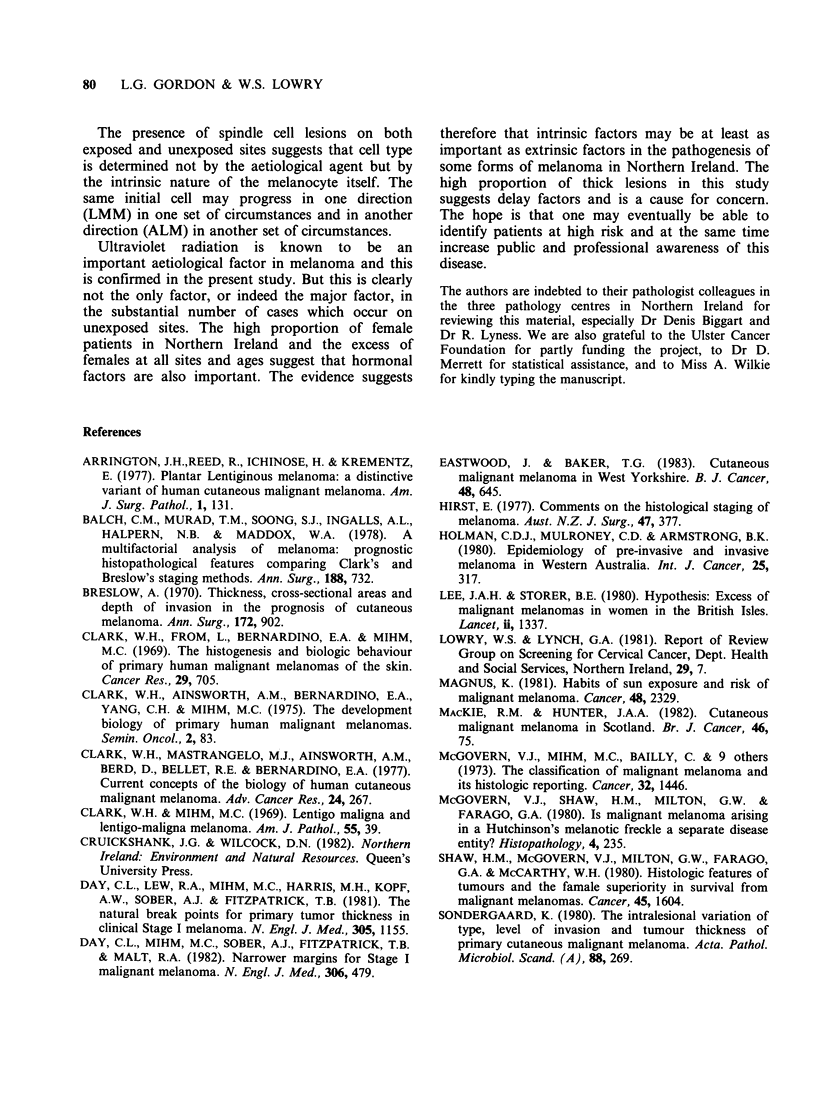# The incidence and pathogenesis of invasive cutaneous malignant melanoma in Northern Ireland.

**DOI:** 10.1038/bjc.1986.11

**Published:** 1986-01

**Authors:** L. G. Gordon, W. S. Lowry

## Abstract

Three hundred and four suspected cases of malignant melanoma diagnosed in Northern Ireland over a 5 year period have been reviewed. Two hundred and forty fulfilled the diagnostic criteria of invasive cutaneous malignant melanoma (CMM) and were accepted as suitable for analysis an incidence of 3.12. The female to male ratio for CMM in this study is 3:1. This excess of female lesions occurs at all major anatomical sites and for all tumour types. There are many thick melanomas in the province, and 67% of cases were greater than 1.7 mm thick. Each tumour type has a distinctive age curve. The implications of these findings are discussed. The evidence suggests that intrinsic factors are as important as extrinsic factors.


*Br. J. Cancer* (1986), **53**, 75–80

### The incidence and pathogenesis of invasive cutaneous malignant melanoma in Northern Ireland

L.G. Gordon & W.S. Lowry

*Department of Oncology, The Queen's University of Belfast, Belfast BT9 7BL, UK.*

**Summary** Three hundred and four suspected cases of malignant melanoma diagnosed in Northern Ireland over a 5 year period have been reviewed. Two hundred and forty fulfilled the diagnostic criteria of invasive cutaneous malignant melanoma (CMM) and were accepted as suitable for analysis an incidence of 3.12. The female to male ratio for CMM in this study is 3:1. This excess of female lesions occurs at all major anatomical sites and for all tumour types. There are many thick melanomas in the province, and 67% of cases were greater than 1.7 mm thick. Each tumour type has a distinctive age curve. The implications of these findings are discussed. The evidence suggests that intrinsic factors are as important as extrinsic factors.

Malignant melanoma is a potentially lethal form of skin cancer. Its incidence and mortality is increasing in most countries studied. The disease is found in a younger age group than most other forms of cancer. Different forms of melanoma may have different patterns of incidence, growth and prognosis.

Apart from Scotland (MacKie & Hunter, 1982) it has been unusual to use the population of an entire country as the basis for a study of melanoma and correlate histopathological parameters with age, sex and site. Northern Ireland is a relatively closed geographical entity with sufficient numbers of cases for analysis. Moreover its limited size allows accessibility to virtually all patients. A detailed histopathological examination of all cases of melanoma referred to the three pathology centres over a 5-year period was therefore undertaken.

The population of Northern Ireland was 1,538,800 at the time of this study. It is situated between 54°N and 56°N latitude. There is a maritime climate and the annual solar radiation incident on a horizontal surface is 1,000 kWh per square meter (Cruikshank & Wilcock, 1982).

### Materials and methods

### *Data accumulation*

One purpose of the study was to identify each new case of primary cutaneous malignant melanoma (CMM) which occurred over a given period of time. This presents a number of difficulties. Cancer is not a notifiable clinical disease and the Northern Ireland Cancer Registry contains incomplete information. For this reason only biopsy proven cases were accepted for the study. There are only three pathology centres in Northern Ireland and all biopsy specimens are submitted to these centres. Through the kind co-operation of the consultant pathologists, the histopathological records of the three centres were completely reviewed for the 5-year period between 1974–1978. The only possible cases of melanoma that might not be included in this survey would presumably be those cases not submitted for biopsy. At a joint melanoma group meeting with general surgeons, plastic surgeons, pathologists, dermatologists and other clinicians in the province, it was considered that very few cases of melanoma would escape histopathological confirmation in Northern Ireland.

Eye lesions, metastases from primaries diagnosed previously, and second excisions were all excluded. Cases of multiple primary melanoma were only considered if the first primary occurred during the period of study.

Three hundred and four cases were thus available for study. Of these, 15 cases of lymph node melanoma had no identifiable primary site and were excluded because no cutaneous melanoma has been found. The remaining 289 cases were histopathologically examined. Eight cases of anal, vaginal and nasal mucosal melanoma were excluded after histopathological examination because no evidence of involvement of stratified squamous epithelium was present. Four doubtful cases of melanoma were excluded following further histological examination. It is generally agreed that non-invasive lesions should be considered separately from invasive malignant melanoma (Hirst, 1977; Holman *et al.*, 1980) and 37 non-invasive lesions were therefore excluded. The remaining 240 cases of CMM formed the basis of this study.

For each case the following additional information was usually available: name, hospital, hospital number, sex, age and anatomical site. The

Correspondence: L.G. Gordon.
Received 21 January 1985; and in revised form, 19 September 1985.

© The Macmillan Press Ltd., 1986

date of birth for seven patients, and anatomical site for nine patients were not available. These unknowns were randomly distributed among the pathology centres. The following specific anatomical sites were considered 'normally exposed' in Northern Ireland: face, neck, scalp, forearm, hand, lower leg (in females only). The remaining sites were classified as 'normally unexposed'.

### *Histopathological assessment*

All 240 cases of CMM were reviewed histopathologically by the authors and the consultant pathologists (see below). Leitz Orthoplan with fitted ocular micrometer was used to examine all specimens. When the diagnosis or classification required additional sections, these were obtained and examined. To perform multiple cuts on all specimens was judged impractical and unnecessary (Sondergaard, 1980). Any parameters not assessable were denoted as 'impossible to ascertain' and are not included in the tables.

The criteria used to assign histopathological type were those given by McGovern *et al.* (1973) and Clark *et al.* (1969, 1975, 1977). Acral-lentiginous melanoma (ALM) is now recognised as a distinct entity, and was used in this study as defined by Arrington *et al.* (1977). Type was assigned without reference to clinical information in most cases. For 227 cases tumour type was ascertainable.

Tumour thickness was measured using an ocular micrometer as described by Breslow (1970). The earlier classification of depth of invasion by dermal level suggested by Clark *et al.* (1969) was also used. In this paper the classification of measurement recommended by Day (1981, 1982) was used. For 231 cases a thickness measurement was ascertainable.

The profile of lesions was classified into three main groups as they appeared in the fixed histopathological specimen: polypoidal/pedunculated; flat; or convex/plateau. Lesions with any degree of histopathological ulceration were assigned the label 'ulceration present'.

The three major cell groups used were: (i) epitheloid cells predominant; (ii) spindle cells predominant; (iii) no predominant cell type.

### *Statistical analysis*

Chi-squared analysis was used to determine relationships between the different variables. When numbers were too small for chi-squared analysis, Fischer's exact probability test was employed. In the few cases where data could not be ascertained for a particular variable, such cases were omitted from chi-squared, exact probability, or age distribution analysis and are not included in the tables. All statements of percentage refer to the percentage of total cases where that variable could be ascertained. All comments made in **Results** have been statistically confirmed.

Age distribution patterns were analysed using the death rate method, the details of which are given in the medical tables of the Annual Review of the Registrar General for England and Wales. All age group differences mentioned are significant at a level of $P<0.05$.

### Results

The incidence of cutaneous malignant melanoma in Northern Ireland is 3.12 per 100,000 population per year. This compares with a figure of only 2.3 reported by the official Northern Ireland Cancer Registry for the same period. For one year alone 41 out of 80 cases were missed by the registry.

Of the 240 genuine cases of primary CMM over the 5 year period, 178 lesions are in females and 62 in males, a ratio of ~3:1. The incidence per 100,000 population per year for females is 4.64 and that for males is 1.63. This female excesss is present for all major anatomical sites, age groups, and tumour types.

### *Sex differences*

Age specific incidences for CMM show a general increase with advancing age in both sexes, but certain differences are apparent (Figure 1). In the 50–59 age group the most dramatic difference in age specific incidence between females and males is seen, *viz.* a female to male ratio of 6:1.

**Figure 1** Age specific incidences of CMM (—) female; (---) male.

**Table I** General anatomical site distribution of CMM.

| | Male | | | Female | | |
|---|---|---|---|---|---|---|
| *Site* | *Number* | *% of male CMM* | *Incidence per 100,000 population per year* | *Number* | *% of female CMM* | *Incidence per 100,000 population per year* |
| Head/neck | 30 | 49 | 0.79 | 45 | 26 | 1.17 |
| Trunk | 11 | 18 | 0.29 | 31 | 18 | 0.81 |
| Hand/arm | 7 | 12 | 0.18 | 18 | 11 | 0.41 |
| Leg/thigh | 7 | 12 | 0.18 | 56 | 33 | 1.46 |
| Foot | 6 | 10 | 0.16 | 20 | 12 | 0.52 |

**Table II** Thickness of CMM.

| | Male | | Female | | Total | |
|---|---|---|---|---|---|---|
| *Thickness in mm* | *Number* | *% of male* | *Number* | *% of female* | *Number* | *% of total* |
| <0.85 | 5 | 9 | 25 | 16 | 32 | 14 |
| | | 18 | | 38 | | 33 |
| 0.85–1.69 | 5 | 9 | 39 | 22 | 44 | 19 |
| 1.70–3.59 | 24 | 41 | 54 | 31 | 78 | 34 |
| | | 82 | | 62 | | 67 |
| ≧3.60 | 24 | 41 | 53 | 31 | 77 | 33 |

The anatomical site distribution also differs between females and males at a level of statistical significance (Table I; $P<0.001$). The absolute number of female lesions exceeds that for males at all general anatomical sites. In absolute terms melanomas of the lower limb are eight times more common in females. There are also more female lesions than male for most of the smaller specific sites even in areas like sole of foot, subungual, perineal skin and nasal mucosa.

Thick lesions are defined in this study as those greater than 1.7 mm. There is a significantly greater proportion of males with thick lesions than females. (Table II; $0.01>P>0.001$). In males, 86% are thick under age 60 and 80% are thick at age 60 and over. In females 52% of female lesions are thick under 60 years and 77% are thick over 60 years.

Tumour type, profile, ulceration, and predominant cell type do not differ significantly between females and males. Even inter-relationships of these variables with age, site and thickness reveal no sex differences, e.g. thick lesions and thin lesions show similar percentages ulcerated in males and females.

*Site*

Anatomical site distribution is described in Table I. Table III shows that there are lesions on 'normally unexposed' sites more than would be expected when compared to the per cent of surface area occupied (chi-squared, observed-expected, $P<0.001$). At the same time the substantial number of melanomas developing on unexposed sites is also noted.

*Thickness*

There is an excess of thick melanomas in the province (Table II). In this study 67% of assessable

**Table III** CMM on exposed and unexposed anatomical sites.

| | Male | | | Female | | |
|---|---|---|---|---|---|---|
| *Exposure* | *Number* | *% of male* | *% of surface area* | *Number* | *% of female* | *% of surface area* |
| Normally exposed | 33 | 54 | 18 | 100 | 59 | 30 |
| Normally unexposed | 28 | 46 | 82 | 70 | 41 | 70 |

cases were >1.7 mm thick (75%>1.5 mm). There is also a significant increase in age specific incidence of thick lesions with each increasing age group.

Almost invariably the following specific sites also have all thick lesions: nostril, vulval skin, anal skin, finger and toe subungual. There is no significant variation in thickness on normally exposed and unexposed sites in either sex.

The distribution of thickness by tumour type is illustrated in Table IV. Even the majority of LMM are thick in both sexes, 67%. ALM is particularly thick with 60% of lesions >4.00 mm.

**Table IV** Thickness distribution by tumour type.

| *Tumour type* | *<1.7 mm* | | *≧1.7 mm* | |
|---|---|---|---|---|
| | *Number* | *% of each type* | *Number* | *% of each type* |
| NM | 21 | 22 | 75 | 78 |
| SSM | 35 | 56 | 27 | 44 |
| ALM | 5 | 20 | 20 | 80 |
| LMM | 14 | 33 | 29 | 67 |

*Type*

Forty-two per cent of all lesions with known type in this study are classified as NM, 27% as SSM, 20% LMM, and 11% ALM. There are significant variations in age-specific incidence patterns for each tumour type (see Table V).

**Table V** Tumour type by age group for CMM (age specific incidences in brackets).

| *Age group* | *Tumour type* | | | |
|---|---|---|---|---|
| | *NM* | *SSM* | *ALM* | *LMM* |
| 20 | 4 (0.14) | 0 (0) | 0 (0) | 0 (0) |
| 20–29 | 8 (0.72) | 4 (0.36) | 0 (0) | 0 (0) |
| 30–49 | 30 (1.74) | 25 (1.45) | 2 (0.12) | 3 (0.17) |
| 50–69 | 28 (1.96) | 24 (1.68) | 15 (1.05) | 15 (1.05) |
| 70+ | 24 (4.32) | 7 (1.26) | 8 (1.44) | 24 (4.32) |

*Profile*

The most common tumour profile is convex/plateau accounting for 44% of those assessable; 41% of lesions are polypoidal/pedunculated; 15% are flat. Tumour profile is not related to tumour type ($0.1 > P > 0.05$). Polypoidal/pedunculated lesions tend to be thicker than other profiles ($P < 0.001$). All male polypoidal lesions and 92% of all female polypoidal lesions are thicker than 1.7 mm.

*Ulceration*

Fifty-eight per cent of all lesions in this study are ulcerated. Not surprisingly there is more tendency to ulceration in thicker lesions ($P = 0.000$). More polypoidal/pedunculated lesions are ulcerated than any other tumour type ($P < 0.001$).

*Cell type*

Tumours with predominantly epitheloid cell type represent 39% of tumours, 36% of lesions contain no predominant cell type and in 25% spindle cells are predominant. Fifty-five per cent of spindle cell lesions are on the head and neck, 18% on the foot and 18% on the trunk. Forty per cent of epitheloid lesions are on the leg. Spindle cell melanomas tend to be deeper than other cell types with 86% of lesions thick ($P = 0.001$). Spindle cell lesions also tend to be more ulcerated than other cell types ($P = 0.041$).

### Discussion

At present cancer is not a notifiable disease in Northern Ireland and the official cancer registry contains incomplete information. Inaccuracies in the registry had already been noted in previous studies of gynaecological cancer in Northern Ireland (Lowry & Lynch, 1981). In the present study discrepancies of over 50% were noted in the registry when the pathology reports were compared with clinical notification. The reasons for these errors are complex and beyond the scope of this paper. However the problems associated with cancer registration are not unique to Northern Ireland and have been found in other studies elsewhere. The present study highlights the importance of obtaining biopsy proven cases for an investigation of this kind. In this connection it is of interest that Australia has recently made cancer a notifiable disease and that the responsibility for notification lies with the pathologist not the clinician in that country.

Possible melanomas not included in this study would therefore be those cases not submitted for biopsy. Such possibilities include cases which were so early they appeared clinically benign at the time of excision, moribund patients with such advanced disease that intervention was considered inappropriate, and patients who refused to consult their doctor or who declined investigation when confronted with the possible diagnosis. Such cases will almost always be missed in any study but the melanoma group considered there would be relatively few instances in this series.

The incidence of 3.12/100,000 is similar to a few other population based studies during the same time period, but is lower than for instance Norway

(Magnus, 1981), and extremely low compared with Australia (Holman *et al.*, 1980).

The three-to-one female to male ratio in Northern Ireland is one of the highest so far reported and suggests that hormonal or other sex-related environmental or genetic factors may be particularly important in this population. In the British Isles a female to male ratio for melanoma of approximately two-to-one has been reported (Lee & Storer, 1980; MacKie & Hunter, 1982) compared with only a small female excess in most other parts of the world. It may be that this marked sex differential is obliterated in other countries by a larger number of predominantly 'solar' cases in those areas.

The high preponderance of female melanoma in Northern Ireland is found for all major anatomical sites, not only the lower limb. It even exists in head and neck disease and for LMM where, because of occupational ultraviolet exposure one might anticipate a high incidence in males. The age distribution in females also suggests that hormonal mechanisms may be important. There is a steady increase in incidence during ages of high oestrogen activity and a levelling off around the time of the menopause. It is at this time, when hormonal changes are at their most profound, that the most dramatic difference between female and male incidence is seen.

Male lesions tend to be thicker than female lesions. This feature reflects tumour progression once the disease is established rather than variation in initial susceptibility to the disease. Thickness differences could be due to longer diagnostic delay in males. Alternatively females may be more susceptible to initiating factors whereas males may have weaker defence mechanisms once the disease is established. Although solar radiation may be an initiating factor in some melanomas, it is unlikely to be responsible for the sex differences in tumour progression as there are no thickness differences between exposed and unexposed sites in males and females. The suggestion that hormonal factors might influence the development of melanoma is also supported by the embryological derivation of the melanocyte from the neural crest. Hormonal influence on tumour growth is supported by the age distribution of thick lesions in men and women. Females develop thicker lesions after the menopause. On the other hand as males grow older lesions have less tendency to become thick.

This study confirms that ultraviolet radiation is a major factor in the aetiology of many melanomas. The high incidence of CMM on the head and neck supports this view as does the high incidence on the female lower leg and other 'normally exposed' sites. However, the relatively high incidence of melanoma on the foot (11% of total) and on other unexposed sites suggests some additional unknown aetiological factors apart from ultraviolet radiation. The distribution and variation of melanoma may therefore depend on both extrinsic and intrinsic factors. The instrinsic factors could possibly include embryological derivation, distribution of naevi, density of melanocytes and other factors.

There is a higher proportion of thick lesions in Northern Ireland than in most other studies, e.g. Shaw *et al.*, 1980; Balch *et al.*, 1978; MacKie & Hunter, 1982; Eastwood & Baker, 1983. This could be due to more aggressive disease but is more likely related to diagnostic delay associated with decreased public and professional awareness of the disease.

LMM, SSM and ALM each have distinct patterns of distribution which suggest that different initiation events and different primary cell types may be involved in each tumour type leading to different patterns of growth. The link between LMM and ultraviolet radiation is well recognised and is similar to that described for squamous and basal cell carcinomas (McGovern *et al.*, 1980; Clark & Mihm, 1969). The age and site distribution of LMM in Northern Ireland supports a cumulative dose ultraviolet aetiology. Although LMM is different in some respects to other types of melanoma it also invades deeply in most cases in Northern Ireland.

ALM also has a lentiginous growth. It differs from LMM however in age distribution, site, and depth of penetration. ALM lesions are particularly thick and it may be that the obscure anatomical sites delay its discovery. LMM lesions are probably precipitated by ultraviolet radiation but the obscure site distribution of ALM suggests some other factor is involved. The variation in age distribution between ALM and LMM also supports this with ALM showing significant increase in age-specific incidence only in the middle-age range.

SSM tends to be less advanced than other forms of melanoma. Although a cohort effect cannot be excluded, the age distribution suggests that this lesion could be related to a single initiating event, or the coincidence of more than one event at a single point in time. The pattern of NM in this study is not distinctive. It may be that NM is often an end-stage lesion that began as another type which has been obliterated by the nodular growth phase.

Tumour profile gives additional information on melanoma independent of tumour type. The increased thickness of polypoidal/pedunculated lesions may represent less restrictions on physical growth as they grow outwardly. It may also indicate more aggressive disease.

The presence of spindle cell lesions on both exposed and unexposed sites suggests that cell type is determined not by the aetiological agent but by the intrinsic nature of the melanocyte itself. The same initial cell may progress in one direction (LMM) in one set of circumstances and in another direction (ALM) in another set of circumstances.

Ultraviolet radiation is known to be an important aetiological factor in melanoma and this is confirmed in the present study. But this is clearly not the only factor, or indeed the major factor, in the substantial number of cases which occur on unexposed sites. The high proportion of female patients in Northern Ireland and the excess of females at all sites and ages suggest that hormonal factors are also important. The evidence suggests therefore that intrinsic factors may be at least as important as extrinsic factors in the pathogenesis of some forms of melanoma in Northern Ireland. The high proportion of thick lesions in this study suggests delay factors and is a cause for concern. The hope is that one may eventually be able to identify patients at high risk and at the same time increase public and professional awareness of this disease.

The authors are indebted to their pathologist colleagues in the three pathology centres in Northern Ireland for reviewing this material, especially Dr Denis Biggart and Dr R. Lyness. We are also grateful to the Ulster Cancer Foundation for partly funding the project, to Dr D. Merrett for statistical assistance, and to Miss A. Wilkie for kindly typing the manuscript.

**References**

ARRINGTON, J.H.,REED, R., ICHINOSE, H. & KREMENTZ, E. (1977). Plantar Lentiginous melanoma: a distinctive variant of human cutaneous malignant melanoma. *Am. J. Surg. Pathol.*, **1**, 131.

BALCH, C.M., MURAD, T.M., SOONG, S.J., INGALLS, A.L., HALPERN, N.B. & MADDOX, W.A. (1978). A multifactorial analysis of melanoma: prognostic histopathological features comparing Clark's and Breslow's staging methods. *Ann. Surg.*, **188**, 732.

BRESLOW, A. (1970). Thickness, cross-sectional areas and depth of invasion in the prognosis of cutaneous melanoma. *Ann. Surg.*, **172**, 902.

CLARK, W.H., FROM, L., BERNARDINO, E.A. & MIHM, M.C. (1969). The histogenesis and biologic behaviour of primary human malignant melanomas of the skin. *Cancer Res.*, **29**, 705.

CLARK, W.H., AINSWORTH, A.M., BERNARDINO, E.A., YANG, C.H. & MIHM, M.C. (1975). The development biology of primary human malignant melanomas. *Semin. Oncol.*, **2**, 83.

CLARK, W.H., MASTRANGELO, M.J., AINSWORTH, A.M., BERD, D., BELLET, R.E. & BERNARDINO, E.A. (1977). Current concepts of the biology of human cutaneous malignant melanoma. *Adv. Cancer Res.*, **24**, 267.

CLARK, W.H. & MIHM, M.C. (1969). Lentigo maligna and lentigo-maligna melanoma. *Am. J. Pathol.*, **55**, 39.

CRUICKSHANK, J.G. & WILCOCK, D.N. (1982). *Northern Ireland: Environment and Natural Resources*. Queen's University Press.

DAY, C.L., LEW, R.A., MIHM, M.C., HARRIS, M.H., KOPF, A.W., SOBER, A.J. & FITZPATRICK, T.B. (1981). The natural break points for primary tumor thickness in clinical Stage I melanoma. *N. Engl. J. Med.*, **305**, 1155.

DAY, C.L., MIHM, M.C., SOBER, A.J., FITZPATRICK, T.B. & MALT, R.A. (1982). Narrower margins for Stage I malignant melanoma. *N. Engl. J. Med.*, **306**, 479.

EASTWOOD, J. & BAKER, T.G. (1983). Cutaneous malignant melanoma in West Yorkshire. *B. J. Cancer*, **48**, 645.

HIRST, E. (1977). Comments on the histological staging of melanoma. *Aust. N.Z. J. Surg.*, **47**, 377.

HOLMAN, C.D.J., MULRONEY, C.D. & ARMSTRONG, B.K. (1980). Epidemiology of pre-invasive and invasive melanoma in Western Australia. *Int. J. Cancer*, **25**, 317.

LEE, J.A.H. & STORER, B.E. (1980). Hypothesis: Excess of malignant melanomas in women in the British Isles. *Lancet*, **ii**, 1337.

LOWRY, W.S. & LYNCH, G.A. (1981). Report of Review Group on Screening for Cervical Cancer, Dept. Health and Social Services, Northern Ireland, **29**, 7.

MAGNUS, K. (1981). Habits of sun exposure and risk of malignant melanoma. *Cancer*, **48**, 2329.

MACKIE, R.M. & HUNTER, J.A.A. (1982). Cutaneous malignant melanoma in Scotland. *Br. J. Cancer*, **46**, 75.

MCGOVERN, V.J., MIHM, M.C., BAILLY, C. & 9 others (1973). The classification of malignant melanoma and its histologic reporting. *Cancer*, **32**, 1446.

MCGOVERN, V.J., SHAW, H.M., MILTON, G.W. & FARAGO, G.A. (1980). Is malignant melanoma arising in a Hutchinson's melanotic freckle a separate disease entity? *Histopathology*, **4**, 235.

SHAW, H.M., MCGOVERN, V.J., MILTON, G.W., FARAGO, G.A. & MCCARTHY, W.H. (1980). Histologic features of tumours and the famale superiority in survival from malignant melanomas. *Cancer*, **45**, 1604.

SONDERGAARD, K. (1980). The intralesional variation of type, level of invasion and tumour thickness of primary cutaneous malignant melanoma. *Acta. Pathol. Microbiol. Scand. (A)*, **88**, 269.