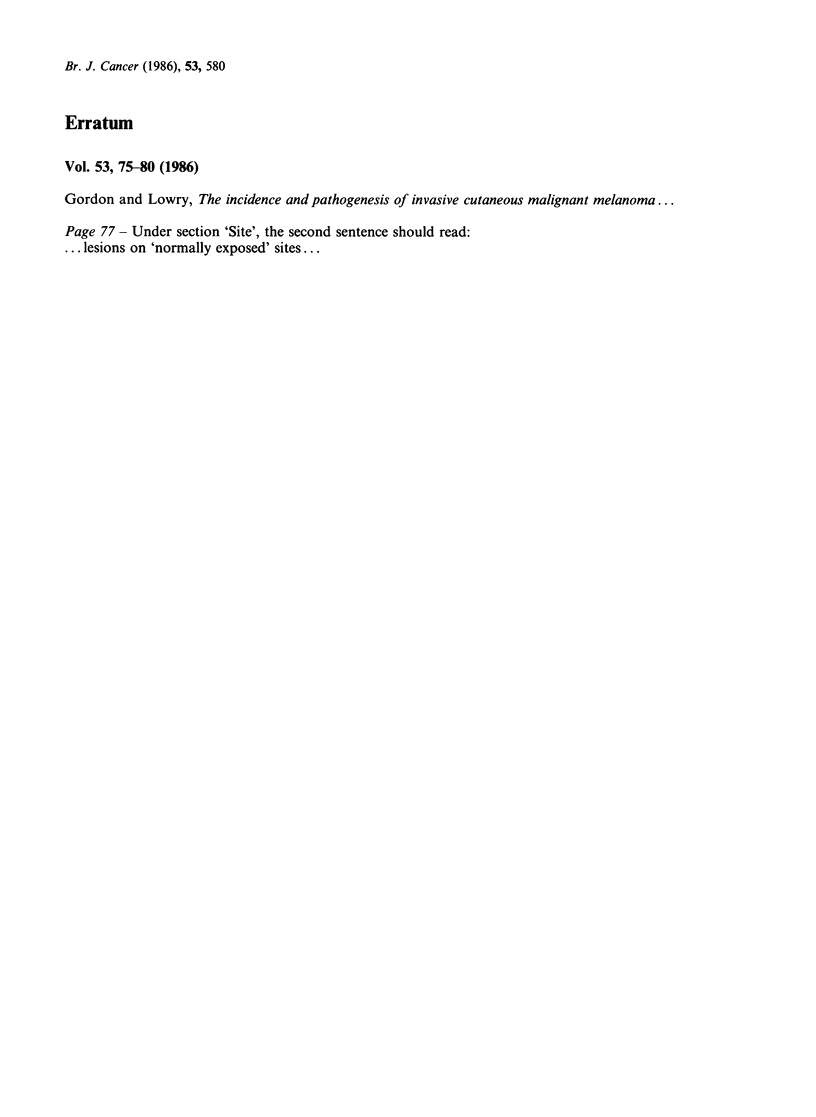# Erratum

**Published:** 1986-04

**Authors:** 


*Br. J. Cancer* (1986), **53,** 580

### Erratum

**Vol. 53, 75–80 (1986)**

Gordon and Lowry, *The incidence and pathogenesis of invasive cutaneous malignant melanoma*...

*Page 77* – Under section 'Site', the second sentence should read:
...lesions on 'normally exposed' sites...